# Peripheral Blood from Rheumatoid Arthritis Patients Shows Decreased T_reg_ CD25 Expression and Reduced Frequency of Effector T_reg_ Subpopulation

**DOI:** 10.3390/cells10040801

**Published:** 2021-04-03

**Authors:** Eunbyeol Go, Su-Jin Yoo, Suyoung Choi, Pureum Sun, Min Kyung Jung, Somin Kwon, Bu Yeon Heo, Yeeun Kim, Ju-Gyeong Kang, Jinhyun Kim, Eui-Cheol Shin, Seong Wook Kang, Jaeyul Kwon

**Affiliations:** 1 Department of Medical Science, College of Medicine, Chungnam National University, Daejeon 35015, Korea; geb0816@gmail.com (E.G.); jdd02287@naver.com (S.C.); reum6133@naver.com (P.S.); xeyk1603@naver.com (B.Y.H.); dpdms1189@hanmail.net (Y.K.); 2 Department of Infection Biology, College of Medicine, Chungnam National University, Daejeon 35015, Korea; 3 Department of Internal Medicine, College of Medicine, Chungnam National University, Daejeon 35015, Korea; sujin428@cnuh.co.kr (S.-J.Y.); jkim@cnuh.co.kr (J.K.); 4 Brain Korea 21 FOUR Project for Medical Science, Chungnam National University, Daejeon 35015, Korea; 5 Laboratory of Immunology and Infectious Diseases, Graduate School of Medical Science and Engineering, Korea Advanced Institute of Science and Technology (KAIST), Daejeon 34141, Korea; jungmk@kaist.ac.kr (M.K.J.); ecshin@kaist.ac.kr (E.-C.S.); 6 Laboratory of Neurogenetics, National Institute on Aging, NIH, Bethesda, MD 20892, USA; kwon.somin@gmail.com; 7 Department of Biological Sciences, Korea Advanced Institute of Science and Technology, Daejeon 34141, Korea; kangju7@kaist.ac.kr; 8 The Center for Epidemic Preparedness, KAIST Institute, Daejeon 34141, Korea; 9 Department of Medical Education, College of Medicine, Chungnam National University, Daejeon 35015, Korea; 10 Translational Immunology Institute, Chungnam National University, Daejeon 35015, Korea

**Keywords:** rheumatoid arthritis, regulatory T cells, CD25, Foxp3, effector regulatory T cells, CD127, naïve regulatory T cells, CD45RA

## Abstract

Rheumatoid arthritis (RA) is a common autoimmune disease characterized by immune cell infiltration of the synovium, leading to the loss of cartilage, bone, and joint function. Although regulatory T (T_reg_) cells are thought to modulate the initiation and progression of RA, a consensus has yet to be reached regarding the function and composition of T_reg_ cells in RA patients. To address these discrepancies, we analyzed not only the total T_reg_ frequency but also that of T_reg_ subpopulations in the peripheral blood of RA patients and healthy controls by flow cytometry. We found that the total T_reg_ population was not significantly different between RA and control subjects. However, the effector T_reg_ cell subgroup, defined as CD45RA^−^CD25^hi^, showed markedly decreased frequency in RA patients. In addition, the total T_reg_ population from RA patients showed a significant decline in the expression of CD25. Both the naïve and effector T_reg_ subgroups also showed marked reduction of CD25 expression in RA patients compared to controls. These data suggest that the decreased frequency of effector T_reg_ cells and overall reduction of CD25 expression in T_reg_ cells in the peripheral blood may be evidence of altered T_reg_ homeostasis associated with RA pathogenesis.

## 1. Introduction

Rheumatoid arthritis (RA) is a common autoimmune disease characterized by the accumulation of inflammatory cells in the joints, leading to chronic synovitis, cartilage and bone damage, and eventual loss of joint function [1,2,3]. Under normal conditions, forkhead box P3 (Foxp3)-positive regulatory T (T_reg_) cells are responsible for maintaining immune tolerance and suppressing potentially harmful autoimmune responses [4]. Depletion of T_reg_ cells has been shown to result in severe autoimmunity in animal models [5]. Several studies have suggested that this homeostasis is similarly disrupted in RA, allowing mononuclear cells and CD4^+^ T cells to infiltrate the synovium unchecked [6,7]. The subsequent release of pro-inflammatory cytokines such as TNF-α can further suppress T_reg_ cell function and exacerbate symptoms [8,9]. However, the frequency and potential dysfunction of T_reg_ cell in RA patients is highly controversial [6,10].

T_reg_ cells are differentiated from CD4^+^ T cells and classically identified by the expression of Foxp3 or IL-2 receptor α chain (CD25). Foxp3 confers suppressive activity to T_reg_ cells, including maintaining expression of CTLA4 [11,12,13], while CD25 allows T_reg_ cells to quickly respond to IL-2 produced by self-reactive T cells [14,15]. Several studies have also suggested using the absence of IL-7 receptor α chain CD127 to distinguish T_reg_ cells, as it has been shown to be inversely correlated with Foxp3 function [16,17]. CD25 has been traditionally used to identify T_reg_ cells [14,15]. The high-affinity IL-2 receptor consists of three distinct subunits designated IL-2Rα (CD25), IL-2Rβ (CD122), and common γ chain (γc; CD132). The trimeric IL-2Rαβγc is typically expressed at high levels by T_reg_ cells, whereas the dimeric IL-2Rβγc is expressed mostly on activated CD8^+^ T cells and NK cells. Foxp3 is another critical marker, as it is considered a master switch gene of the T_reg_ cell lineage and orchestrates its various cellular programs [11,12]. In addition, absent or low cell surface expression of CD127, the α chain of the interleukin-7 receptor, in combination with CD25 expression, has been shown to distinctively distinguish T_reg_ cells from effector T cells expressing high levels of CD127 in humans [16,17]. CD127 expression has been further shown to correlate inversely with Foxp3 expression. These markers are not exclusive to T_reg_ cells, however, as activated CD4^+^ T cells without suppressive activity have been also shown to be Foxp3^+^CD25^+^ and CD25^+^CD127^−^ [18,19]. As such, detecting T_reg_ cells and identifying their role in RA may be significantly more intricate than previously thought.

CD25 is a part of the high-affinity IL-2 receptor complex constitutively expressed in T_reg_ cells and shown to play a critical role in the generation and function of T_reg_ cells [20,21]. Peripheral survival and expansion of T_reg_ cells may additionally require appropriate IL-2/IL-2R signaling [22,23,24]. Several genes within the IL-2/IL-2R pathway (e.g., *IL2*, *IL2RA*, *IL2RB,* and *PTPN2*) may influence an individual’s susceptibility to human autoimmune diseases, with evidence to suggest that these genes exert their effects by altering T_reg_ cell frequency or function [25,26]. Of note, a candidate gene study identified an association between RA and *IL2RA*, which encodes CD25 [26,27,28]. Low CD25 expression was identified as a phenotypic characteristic of T_reg_ cells in the peripheral blood of patients with autoimmune diseases such as type I diabetes [29] and systemic lupus erythematosus [30]. Gene polymorphisms of the proteins in the IL-2/IL-2R pathway may similarly exert their influence on RA risk via changes to the frequency or phenotype of T_reg_ cells. Recently, low-dose IL-2 therapy was applied as a new clinical approach for autoimmune diseases that takes advantage of the selective activity of low-dose IL-2 on T_reg_ cells [31,32]. Several studies have demonstrated that low-dose IL-2 therapy can successfully overcome T_reg_ cell deficiency and increase the ratio of T_reg_ to effector T cells in patients with RA and other autoimmune diseases [33,34,35].

Functional deficiency in the T_reg_ compartment has long been considered a cause of autoimmune diseases such as RA [36,37,38]. However, previous literature has been unable to reach a consensus as to the specific role of T_reg_ cells in peripheral immune tolerance in RA, or even the frequency of T_reg_ cells in peripheral blood of RA patients [7,39]. Interestingly, various therapies currently approved for RA, such as methotrexate, adalimumab, and tocilizumab, have been shown to restore the frequency and function of T_reg_ cells [40,41,42,43]. Given the inconsistent reports regarding the frequency and potential dysfunction of T_reg_ cells in RA patients and accumulating evidence that human T_reg_ cells are heterogeneous in phenotype and function, we sought to offer a more precise assessment of T_reg_ cells in RA patients by examining their subpopulations. Previous studies have delineated T_reg_ cells into naïve (Fr. I), effector (Fr. II), and non-suppressive T_reg_ subsets (Fr. III) based on CD25, Foxp3, and CD45RA expression in CD4^+^ T cells [44]. In this study, we examined these T_reg_ subgroups in the peripheral blood of RA patients and healthy control subjects. We report that the total frequency of T_reg_ cells did not change significantly in RA patients and healthy controls. However, the frequency of effector T_reg_ cells (Fr. II) was markedly reduced in RA patients. Furthermore, CD25 expression in T_reg_ cells was significantly decreased in RA patients. Our observation may be instrumental in understanding the function and adaptive processes of T_reg_ cells in RA disease progression.

## 2. Materials and Methods

### 2.1. Human Subjects

Blood samples were collected from RA patients and healthy adult volunteers under IRB approval (IRB file No. CNUH 2015-10-052, approval date 2015-10-28) from Chungnam National University Hospital (Republic of Korea). RA patients (*n* = 13) were diagnosed according to the 2010 American College of Rheumatology criteria. Patients were divided by RA disease activity according to the clinical parameter Disease Activity Score 28 (DAS28) [3,45]. Healthy adult volunteers (*n* = 13) were enrolled in this study and had no acute or chronic inflammatory or infectious disease, ongoing thrombosis, or neoplasia. Subject characteristics are provided in Table S1. All studies were performed in accordance with the Declaration of Helsinki.

### 2.2. PBMC Isolation

PBMC (peripheral blood mononuclear cells) were obtained from whole blood using lymphocyte separation medium (Corning) by density gradient centrifugation.

### 2.3. Flow Cytometric Analysis

To distinguish live and dead cells, PBMC were stained with live/dead fixable stain dye (Life technologies). After PBS washing, cells were incubated with FITC-CD3 (BD Biosciences), PerCP-Cy5.5-CD4 (BD Biosciences), BV421-CD25 (BD Biosciences), APC-CD127 (Biolegend), and PE-Cy7-CD45RA (BD Biosciences). Cells were then fixed and permeabilized with Foxp3/Transcription Factor Staining Buffer Set (eBioscience) and further stained with PE-Foxp3 (BD Biosciences). Cells were analyzed with a FACSCanto II flow cytometer (BD Biosciences), and data were processed with FlowJo software (Tree Star, OR, USA).

### 2.4. Statistical Analysis

Data were analyzed by Mann–Whitney test using GraphPad Prism (v7.02, GraphPad). Dot plot data in the figures were presented as median with interquartile range, and data in the tables are presented as median values with minimum to maximum range. *p* < 0.05 was considered statistically significant.

## 3. Results

### 3.1. Total Frequency of T_reg_ Cells in Peripheral Blood Did Not Show Significant Difference between RA and Control Subjects

**Figure 1:**
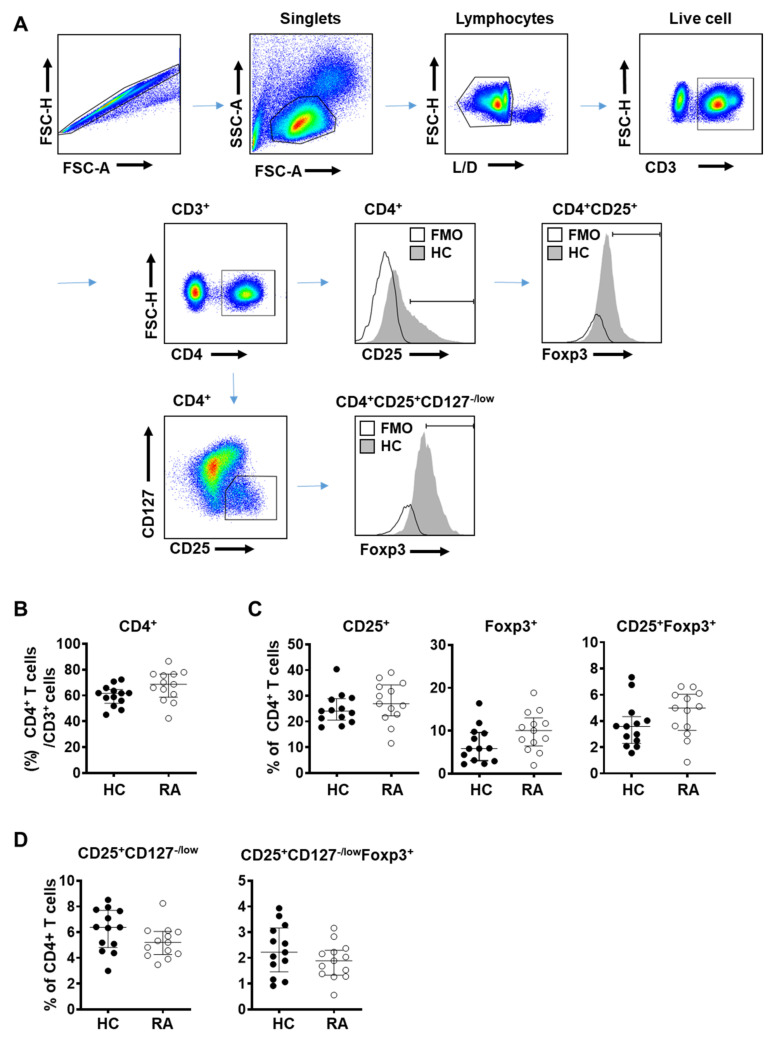
Frequency of regulatory T (T_reg_) cells did not change in patients with rheumatoid arthritis (RA). Blood samples were collected from healthy donor (HC, *n* = 13) and rheumatoid arthritis patients (RA, *n* = 13) and analyzed by flow cytometry. (**A**) Flow cytometry gating scheme of T_reg_ subpopulations in human peripheral blood mononuclear cells (PBMC). FMO (fluorescence minus one control); HC (healthy control). Percentage of (**B**) CD4^+^ T cells among CD3^+^ T lymphocytes in PBMC, (**C**) CD25^+^, Foxp3^+^, or CD25^+^Foxp3^+^ T_reg_ cells among CD4+ T cells, and (**D**) CD25^+^CD127^−/low^, or CD25^+^CD127^−/low^ Foxp3^+^ T_reg_ cells among CD4^+^ T cells. Data from individual subjects were presented with the median values. Statistical differences were calculated by Mann–Whitney test.

To assess the total T_reg_ population in RA patients, we defined T_reg_ cells using molecular markers such as CD25, CD127, or Foxp3 and analyzed their proportion among CD4^+^ T cells in the peripheral blood of RA patients and healthy donors (Figure 1A) [46,47]. Disease severity of the RA subjects was in the range of remission to moderate stages, according to the clinical parameter Disease Activity Score 28 (DAS 28) (Table S1) [3]. Given the limited number of subjects (*n* = 13) in the study, all data were analyzed with a non-parametric test (Mann–Whitney test), although the majority displayed normal distribution.

The proportion of CD4^+^ T cells among CD3^+^ lymphocytes was similar between RA patients and control subjects (Figure 1B). Frequency of T_reg_ cells among CD4+ T cells defined using CD25^+^ alone, Foxp3^+^ alone, and CD25^+^Foxp3^+^ was slightly elevated in RA patients compared to controls but did not reach statistical significance (Figure 1C). When T_reg_ cells were defined as CD4^+^CD25^+^CD127^−/low^ or CD4^+^CD25^+^CD127^−/low^Foxp3^+^, their frequency among CD4^+^ T cells showed a decreasing tendency in RA patients but was not statistically different compared to controls (Figure 1D).

A proportion of early activated conventional T cells has been suggested to show a transient change in expression level of certain cell surface markers, mainly Foxp3, CD127 and CD25, which could be a hurdle to a precise identification of T_reg_ cells. Given that RA patients may also have a greater proportion of activated conventional T cells, accurately assessing the total T_reg_ population in RA patients may be challenging.

### 3.2. Frequency of Effector T_reg_ Cells Is Decreased in the Peripheral Blood from RA Patients

**Figure 2:**
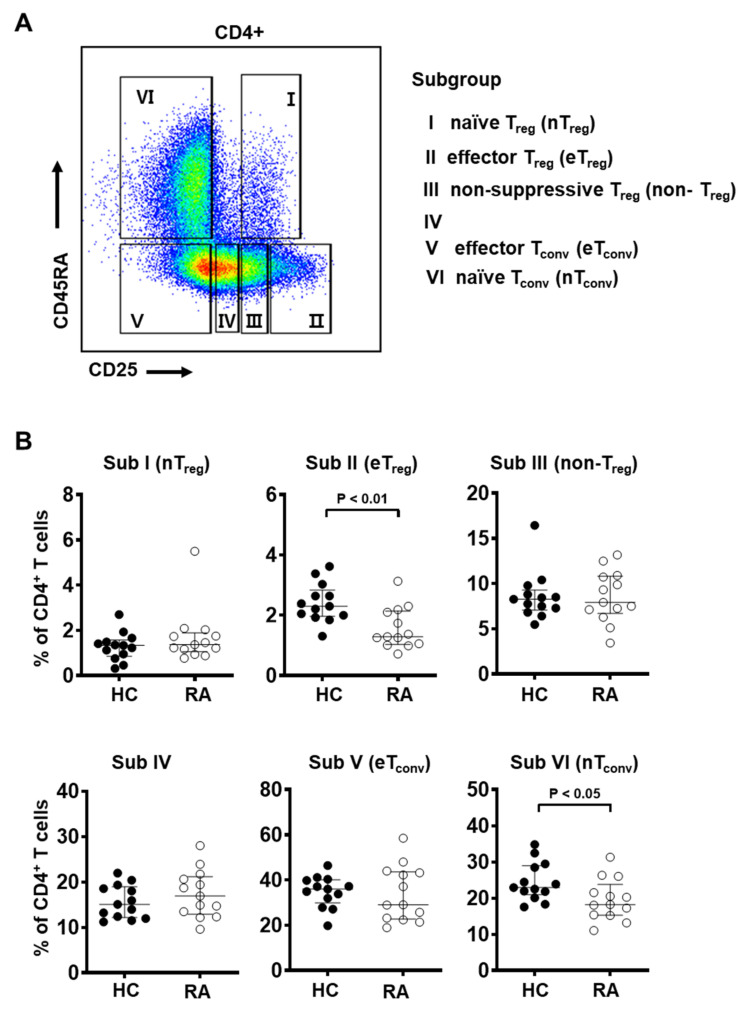
The frequency of effector T_reg_ subpopulations is decreased in the peripheral blood from RA patients. (**A**) Gating strategy to identify six subgroups of CD4^+^ T cells based on the expression levels of CD25 and CD45RA: CD25^int^CD45RA^+^ cells (Subgroup I, naïve T_reg_), CD25^hi^CD45RA^−^ cells (Subgroup II, effector T_reg_), CD25^int^CD45RA^−^ cells (Subgroup III, non-suppressive T_reg_), CD25^low^CD45RA^−^ cells (Subgroup IV), CD25^−^CD45RA^−^ cells (Subgroup V, effector T_conv_), and CD25^−^CD45RA^+^ cells (Subgroup VI, naïve T_conv_). (**B**) Frequency of each subgroup among CD4^+^ T cells of PBMC from healthy controls (HC, *n* = 13) and RA patients (*n* = 13). Statistical differences between HC and RA were calculated by Mann–Whitney test and presented as *p*-value.

Previously several reports on the frequency of T_reg_ cells in the peripheral blood of RA patients have provided conflicting results. Our data did not show a statistically significant difference in the total T_reg_ population between RA patients and healthy controls (Figure 1). As a way to consider heterogeneity of the T_reg_ compartment and analyze the property of T_reg_ subgroups, we introduced the CD45RA marker to discriminate between antigen-experienced T_reg_ (e.g., CD45RA^−^) and naïve T_reg_ (e.g., CD45RA^+^) cells (Figure 2A).

Based on markers CD25 and CD45RA, previous studies have identified distinct T cell subgroups with differential Foxp3 expression and suppressive capacity [44,48,49]. The subgroups are as follows: CD25^int^CD45RA^+^ cells (Subgroup I, naïve/resting T_reg_), CD25^hi^CD45RA^−^ cells (Subgroup II, activated/effector T_reg_), CD25^int^CD45RA^−^ cells (Subgroup III, non-suppressive T_reg_), CD25^low^CD45RA^−^ cells (Subgroup IV), CD25^−^CD45RA^−^ cells (Subgroup V, effector T_conv_), and CD25^−^CD45RA^+^ cells (Subgroup VI, naïve T_conv_). Among these, subgroups I, II, and III were Foxp3^+^ and the degree of Foxp3 expression was found to be proportional to CD25 expression. Specifically, CD25^int^CD45RA^+^ cells (Subgroup I) and CD25^int^CD45RA^−^ cells (Subgroup III) were Foxp3^low^, but CD25^hi^CD45RA^−^ cells (Subgroup II) were Foxp3^hi^.

Using these groupings, we found that the frequency of effector T_reg_ cells (Subgroup II, CD25^hi^CD45RA^−^) was markedly decreased in RA patients compared to healthy controls (Figure 2B). Interestingly, naïve T_conv_ cells (Subgroup VI, CD25^−^CD45RA^+^) were decreased in RA patients compared to healthy controls, suggesting that conventional T cells are more likely to be in an activated state in RA patients given the chronic inflammatory condition (Figure 2B). The other T_reg_ subgroups, naïve T_reg_ (Subgroup I, CD25^int^CD45RA^+^) and non-suppressive T_reg_ cells (Subgroup III, CD25^int^CD45RA^−^ cells), did not show statistically significant differences between RA patients and controls.

We performed the same analysis of CD25/CD45RA subgroups among T_reg_ cells defined as CD25^+^CD127^−/low^ (Figure S1A,B) or CD25^+^CD127^−/low^Foxp3^+^ (Figure S1A,C). In both populations, the frequency of effector T_reg_ cells (Subgroup II, CD25^hi^CD45RA^−^) was markedly decreased in RA patients (Figure S1B,C). The proportion of effector T_reg_ cells was similarly decreased among CD25^+^Foxp3^+^ T_reg_ cells (Figure S2) in RA patients. These data suggest a significant decrease in the proportion of effector T_reg_ cells among the total Foxp3^+^ T_reg_ population in the peripheral blood of RA patients.

### 3.3. CD25 Expression Is Significantly Reduced in T_reg_ Cells in the Peripheral Blood of RA Patients

The decreased frequency of effector T_reg_ cells may be due to altered T_reg_ homeostasis in RA patients. IL-2/IL-2R signaling has been recently shown to be critical for homeostasis of T_reg_ cells [22,23]. Furthermore, *IL2RA* coding for CD25 is a known susceptibility factor for RA [26,27,28], and RA patients have been shown to have mild but significant decreases in circulating IL-2 [50,51]. Activated CD4^+^ T cells from RA patients are also known to produce less IL-2 [52,53]. Given that abnormalities of IL-2/IL-2R signaling pathways may lead to the breakdown of self-tolerance mechanisms in RA, we examined whether expression of IL-2Rα (CD25) in T_reg_ cells was altered in RA patients.

**Figure 3:**
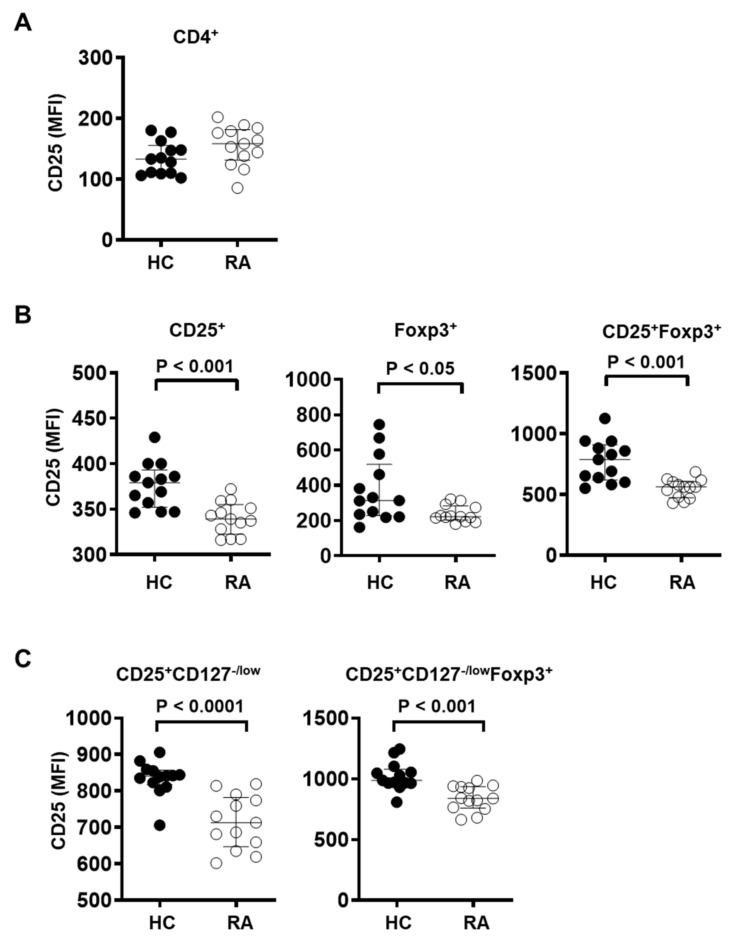
T_reg_ cells from RA patients show decreased CD25 expression. CD25 expression level was measured by flow cytometry in (**A**) CD4^+^ T cells, (**B**) CD4^+^CD25^+^, CD4^+^Foxp3^+^, or CD4^+^CD25^+^Foxp3^+^ T_reg_ cells, and (**C**) CD4^+^CD25^+^CD127^−/low^, CD4^+^CD25^+^CD127^−/low^Foxp3^+^ T_reg_ cells in PBMC from healthy controls (HC, *n* = 13) and RA patients (RA, *n* = 13). Statistical differences were calculated by Mann–Whitney test.

Among T_reg_ cells defined as CD25^+^Foxp3^+^, CD25^+^CD127^−/low^, or CD25^+^CD127^−/low^Foxp3^+^, CD25 expression was significantly reduced in RA patients compared to healthy controls (Figure 3B,C). CD25^+^ and Foxp3^+^ T_reg_ cells from RA patients also displayed reduced expression of CD25 (Figure 3B), although CD25 expression among total CD4^+^ T cells was similar between RA patients and control subjects (Figure 3A). These data suggest that the IL-2R signaling pathway in T_reg_ cells may be impaired in RA patients.

### 3.4. CD25 Expression in Naïve and Effector T_reg_ Cells Is Significantly Reduced in the Peripheral Blood of RA Patients

**Figure 4:**
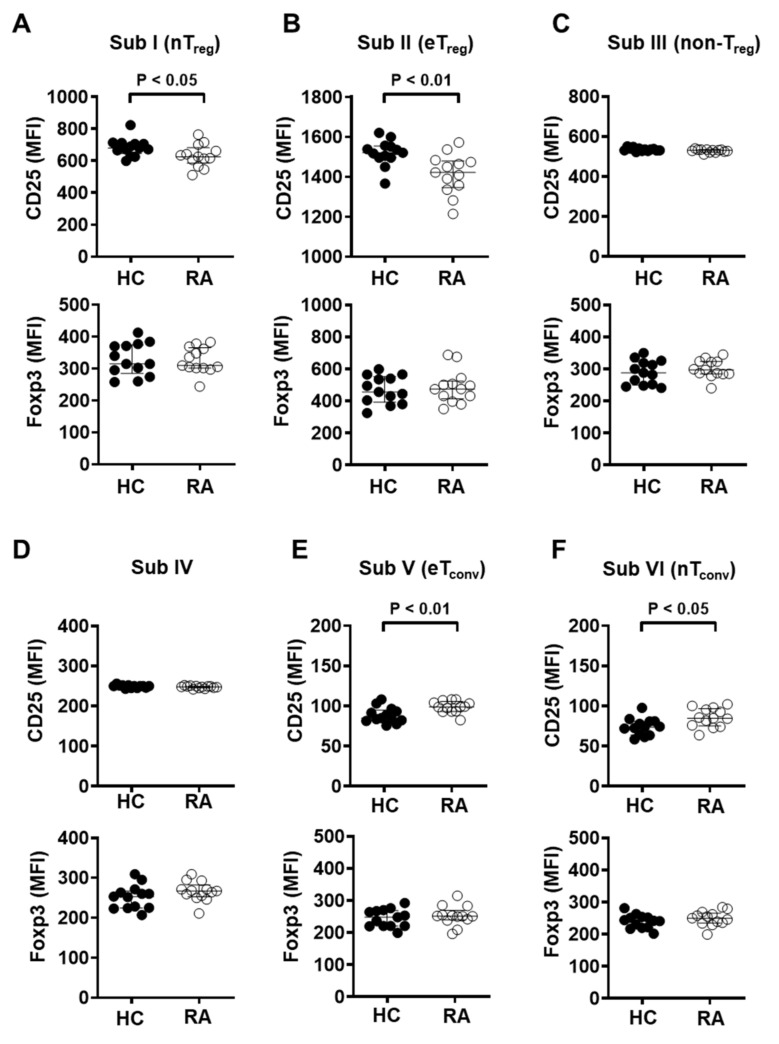
Naïve and effector T_reg_ subpopulations from RA patients showed significantly reduced CD25 expression. Expression of CD25 and Foxp3 was measured by flow cytometry in each CD4^+^ T cell subgroup from healthy controls (HC, *n* = 13) and RA patients (RA, *n* = 13). Subgroups were as follows: (**A**) I (CD25^int^CD45RA^+^ cells), (**B**) II (CD25^hi^CD45RA^−^ cells), (**C**) III (CD25^int^CD45RA^−^ cells), (**D**) IV (CD25^low^CD45RA^−^ cells), (**E**) V (CD25^−^CD45RA^−^ cells), and (**F**) VI (CD25^−^CD45RA^−^ cells). Statistical differences were calculated by Mann–Whitney test.

Given the decreased CD25 expression in the total T_reg_ population, it was then measured in the six T_reg_ subgroups. Expression levels of CD25 in naïve T_reg_ (Subgroup I) or effector T_reg_ (Subgroup II) cells were markedly decreased in RA patients (Figure 4A,B). However, naïve and effector conventional CD4^+^ T cells, Subgroup VI and Subgroup V, respectively, showed significantly increased CD25 expression in RA patients compared with those of heathy controls, suggesting that conventional CD4^+^ T cells maintain an activated status in RA patients (Figure 4E,F). In all subgroups, Foxp3 expression did not show significant differences between RA patients and controls (Figure 4A–F).

Within T_reg_ cells defined as CD25^+^Foxp3^+^ (Figure S3A), CD25 expression was markedly decreased in naïve T_reg_ (Subgroup I), effector T_reg_ (Subgroup II), or non-suppressive T_reg_ (Subgroup III) cells in RA patients. Conversely, Foxp3 expression was significantly elevated among naïve T_reg_ and effector T_reg_ cells in RA patients. Within CD25^+^CD127^−/low^ T_reg_ cells (Figure S3B), naïve T_reg_ and effector T_reg_ cells also showed a significant decrease in CD25 expression in RA patients, with increased Foxp3 expression in effector T_reg_ cells in RA patients. In addition, within CD25^+^CD127^−/low^Foxp3^+^ T_reg_ cells (Figure S3C), CD25 expression was reduced among effector and non-suppressive T_reg_ cells in RA patients, while Foxp3 expression was increased in Subgroups I, II, or III. A previous study had similarly shown that DMARD-exposed RA patients have higher Foxp3 expression compared to healthy controls [40]. Overall, naive and effector T_reg_ cells displayed decreased CD25 expression in RA patients relative to controls with a concomitant increase in Foxp3. These data suggest that abnormalities in IL-2R signaling of T_reg_ cells in RA patients may lead to a lower frequency of effector T_reg_ cells.

## 4. Discussion

In this study, we analyzed the frequency of total circulating T_reg_ cells and their subpopulations in the peripheral blood of RA patients and healthy controls. We showed that despite using well-validated markers, the proportion of the total T_reg_ cell population, such as CD25^+^Foxp3^+^ and CD25^+^Foxp3^+^ CD127^−/low^, in CD4^+^ T cells did not show any significant change in the peripheral blood of RA patients. The frequency of total T_reg_ cells in RA patients has been highly controversial [6,10]. However, our analysis of T_reg_ subgroups among CD4^+^ T cells and the total T_reg_ population consistently demonstrated a significant reduction of effector T_reg_ cells (CD25^hi^CD45RA^−^) in RA patients. Furthermore, expression levels of CD25, the α chain of the IL-2 receptor, constitutively expressed at the T_reg_ cell surface membrane, was markedly decreased among T_reg_ cells in RA patients, while Foxp3 expression in RA patients was significantly higher compared with that in healthy controls. In contrast, subpopulations of conventional CD4^+^ T cells showed a significant increase of CD25 expression in RA patients. These data suggest that low CD25 expression associated with abnormal IL-2/IL-2R signaling may lead to reduced frequency of effector T_reg_ cells and serve as a phenotypic characteristic of T_reg_ cells in peripheral blood of RA patients.

While it is generally agreed that the frequency of T_reg_ cells is increased in the synovial fluid of RA patients, similar studies of the peripheral blood reported largely inconsistent results [6]. Some studies showed an increase in circulating T_reg_ cells in RA patients [54,55,56] while others reported no change [18,57], and still others showed an increase [58,59]. These discrepancies have been attributed to a lack of consensus on T_reg_ markers [6,7]. Indeed, in our study alone we observed an increasing trend of CD25^+^Foxp3^+^ T_reg_ cells in RA patients, but a decreasing trend in CD25^+^CD127^−/low^Foxp3^+^ T_reg_ cells in RA patients (Figure 1). In addition to inconsistent detection strategies, the functional and phenotypic heterogeneity of T_reg_ cells may further obscure their role in RA [44,48]. Here we report that certain T_reg_ subpopulations among PBMC show a strong association with RA. The effector T_reg_ subgroup with suppressive functions (CD25^hi^CD45RA^−^) was significantly decreased in RA patients, while the frequency of naïve T_reg_ (CD25^int^CD45RA^+^) or non-suppressive T_reg_ (CD25^int^ CD45RA^−^) cells did not show a significant difference (Figure 2B).

A recent study used a robust statistical test for disease associations with single cell data, called as MASC (mixed-effects modeling of associations of single cells), to show that the frequency of two T_reg_ subsets expressing high levels of CD25 and Foxp3 was significantly reduced among resting CD4^+^ memory T cells in the peripheral blood of RA patients [60]. Furthermore, increased T_reg_ frequencies in the synovial fluid of RA patients were reported to be due to an increased number of Subgroup III T_reg_ cells (CD25^int^CD45RA^−^) with little suppressive activity and even some pro-inflammatory properties [56]. Notably, there was no observed difference in naïve and effector T_reg_ cells between the synovial fluid and peripheral blood of RA patients. These observations suggest that the T_reg_ compartment may be functionally impaired in RA patients. In line with our findings, this dysfunction may be at least in part attributable to the low frequency of effector T_reg_ cells (CD25^hi^CD45RA^−^) and manifest as an inability to suppress autoreactive T cells.

The effector T_reg_ subgroup has been previously described to have strong suppressive capacity [44,48]. The functional consequences of fewer effector T_reg_ cells in RA patients and the mechanisms responsible remain to be elucidated. Previous studies showed that IL-2 is essential for the optimal development, survival, and function of T_reg_ cells [20,21,22,23] and RA patients were shown to have lower serum IL-2 and reduced IL-2 production upon T cell activation [50,51,52,53]. We further show that expression of CD25, the α chain of the IL-2 receptor, was markedly reduced among T_reg_ cells in RA patients. The IL-2 receptor α chain (CD25) is essential for the high-affinity IL-2 receptor complex, allowing T_reg_ cells to respond to very low concentrations of IL-2 [21,61]. Given that appropriate IL-2 signaling is needed for the expression of pro-survival genes such as *BCL2* and *BCL2L1* (coding BCL-XL) and overall survival of T_reg_ cells [62,63,64,65,66], the degree of CD25 expression in T_reg_ cells may be critical to their homeostasis and survival [29,30]. Highlighting its importance, several genes involved in IL-2/IL-2R signaling such as *IL-2-IL21*, *CTLA4*, *IL2RA*, *IL2RB*, *CD83*, *PTPN2*, and *CCR6* were included in a list of RA risk gene loci identified to date in the transethnic genome-wide association study (GWAS) meta-analysis of RA [25,26]. In addition, *CTLA4* and *IL2RA* were found to be down-regulated among RA patients in an integrated analysis of GWAS and eQTL data [26]. These reports suggest that the reduction of effector T_reg_ cells in RA patients may be linked to impaired IL-2/IL-2R signaling, due to both decreased expression of IL-2Rα (CD25) in T_reg_ cells and the limited availability of IL-2 from reduced IL-2 production by autoreactive conventional CD4^+^ T cells. Interestingly, several current therapies for RA have been reported to expand the T_reg_ compartment [40,41]. Among RA patients, an increased frequency of CD4^+^CD25^hi^ T_reg_ cells has been associated with responders to anti-TNF-α therapy compared to non-responders [42]. Similarly, CD25^+^Foxp3^+^ T_reg_ cells in RA patients were reported to be expanded by anti-IL-6R blockade. Recently, a low-dose IL-2 therapy induced the expansion of the T_reg_ compartment in autoimmune disease patients including those with RA [33].

Our findings highlight the heterogeneity of T_reg_ cells in the peripheral blood and suggest that the effector T_reg_ subset in particular may be critical to RA pathogenesis. Due to limited IL-2/IL-2R signaling, effector T_reg_ cells may be unable to apply appropriate regulatory function against the rampant inflammation seen in RA. As one way of understanding the heterogeneity of T_reg_ cells in the peripheral blood, distinct T cell subgroups with differential Foxp3 expression and suppressive capacity were classified based on markers CD25 and CD45RA [42,44]. We can classify the T cells as CD25-positive or -negative based on flow cytometric analysis of cell surface CD25 expression. CD25^−^CD45RA^−^ cells (Subgroup V, effector T_conv_), and CD25^−^CD45RA^+^ cells (Subgroup VI, naïve T_conv_) were identified as CD25-negative. CD25-positive groups include CD25^low^CD45RA^−^ cells (Subgroup IV), CD25^int^CD45RA^−^ cells (Subgroup III, non-suppressive T_reg_), CD25^int^CD45RA^+^ cells (Subgroup I, naïve/resting T_reg_), and CD25^hi^CD45RA^−^ cells (Subgroup II, activated/effector T_reg_). Among effector T_conv_ and naïve T_conv_ cells, CD25 expression was higher in RA patients than in healthy controls. In contrast, both the total population of regulatory T cells and its subgroups demonstrated significantly reduced CD25 expression in RA patients compared to controls. The generalizability of our observations is limited by the relatively small sample size. It will be important to characterize our findings in a larger group of RA patients with careful stratification. Although this study focused on assessing the variations in frequency of T_reg_ subsets, to fully understand the role of T_reg_ cells in RA and their potential as therapeutic targets, phenotypic characteristics and the suppressive function of T_reg_ cells must also be elucidated. Further study is warranted on the mechanisms of impaired effector T_reg_ function in RA pathogenesis and its potential as a biomarker for the basis of therapeutic development [49].

## Data Availability

The data used to support the findings of this study are available from the corresponding author upon request.

## Supplementary Materials

The following are available online at https://www.mdpi.com/article/10.3390/cells10040801/s1. Table S1. Characteristics of controls and RA subjects; Figure S1. Frequency of effector T_reg_ cells among the total T_reg_ cells defined as CD25^+^CD127^−/low^ or CD25^+^CD127^−/low^Foxp3^+^ is decreased in PBMC of RA patients; Figure S2. Frequency of effector T_reg_ cells among CD25^+^Foxp3^+^ T_reg_ cells is decreased in PBMC of RA patients; Figure S3. Naïve and effector T_reg_ cells from RA patients show decreased CD25 expression.
