# Supplementary material for: Peripheral Blood from Rheumatoid Arthritis Patients Shows Decreased Treg CD25 Expression and Reduced Frequency of Effector Treg Subpopulation
Source: Cells. 2021 Apr 3;10(4):801. doi: 10.3390/cells10040801 (PMC8067140; doi:10.3390/cells10040801)
Supplement: Supplementary file 1 [file cells-10-00801-s001.pdf]

**Figure S1.**

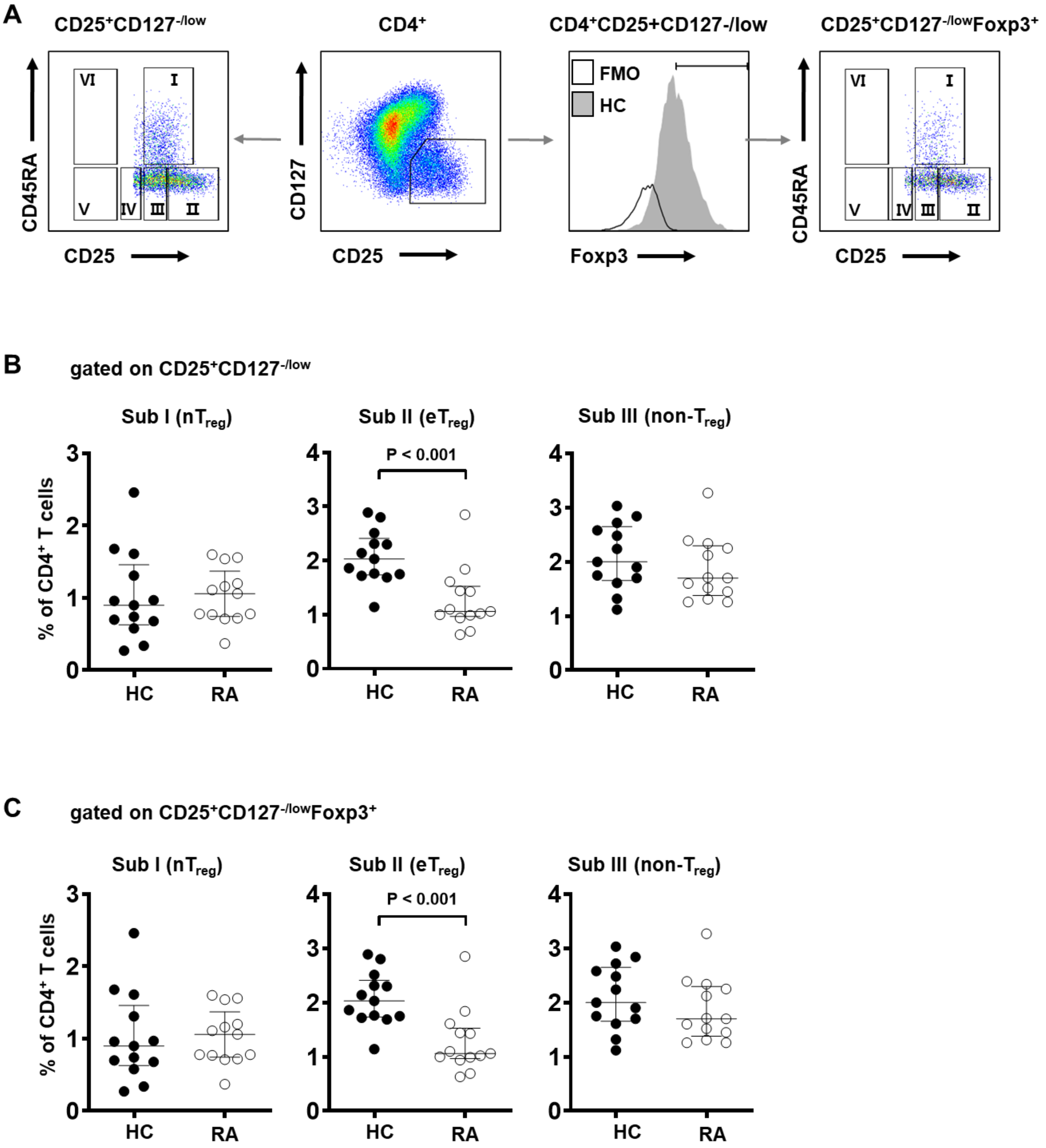


**Figure S1.** Frequency of effector $T_{reg}$ cells among total $T_{reg}$ cells defined as $CD25^{+}CD127^{-/low}$ or $CD25^{+}$ $CD127^{-/low}Foxp3^{+}$ is decreased in PBMCs of RA patients. Blood samples were collected from healthy donors (HC, n = 13) and RA patients (RA, n = 13) and $T_{reg}$ cell subpopulations were analyzed by flow cytometry. (A) Representative plots show the gating strategy used to identify subgroup I ($CD25^{int}$ $CD45RA^{+}$ cells), II ($CD25^{hi}CD45RA^{-}$ cells), and III ($CD25^{int}CD45RA^{-}$ cells) in $CD25^{+}CD127^{-/low}$ or $CD25^{+}$ $CD127^{-/low}Foxp3^{+}$ $T_{reg}$ cells by flow cytometry. (B) Frequency of subgroup I, II, and III in $T_{reg}$ cells defined by $CD25^{+}CD127^{-/low}$. (C) Frequency of subgroup I, II, and III in $T_{reg}$ cells defined by $CD25^{+}$ $CD127^{-/low}$ $Foxp3^{+}$. Statistical differences were calculated by Mann-Whitney test.

**Figure S2.**

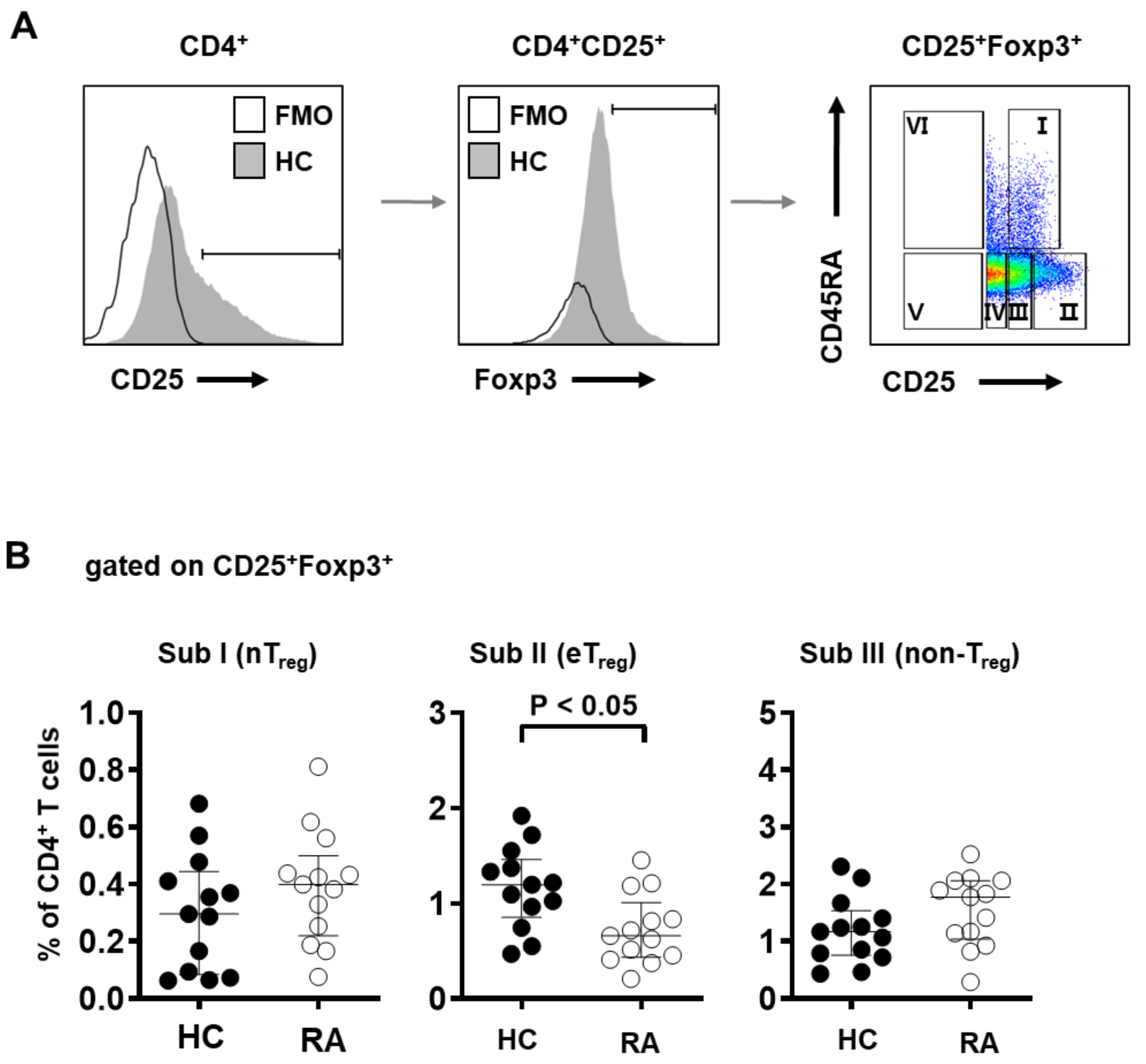


**Figure S2.** Frequency of effector $T_{reg}$ cells among $CD25^{+}Foxp3^{+}$ $T_{reg}$ cells is decreased in PBMCs of RA patients. (A) Representative plots show the gating strategy used to identify subgroup I ($CD25^{int}CD45RA^{+}$ cells), II ($CD25^{hi}CD45RA^{-}$ cells), and III ($CD25^{int}CD45RA^{-}$ cells) in $CD25^{+}Foxp3^{+}$ Treg cells by flow cytometry. FMO (fluorescence minus one control); HC (healthy control). (B) Frequency of subgroup I, II, and III in $CD25^{+}Foxp3^{+}$ $T_{reg}$ cells of PBMCs from healthy controls (HC, n = 13) and RA patients (RA, n = 13). Statistical differences were calculated by Mann-Whitney test.

**Figure S3.**

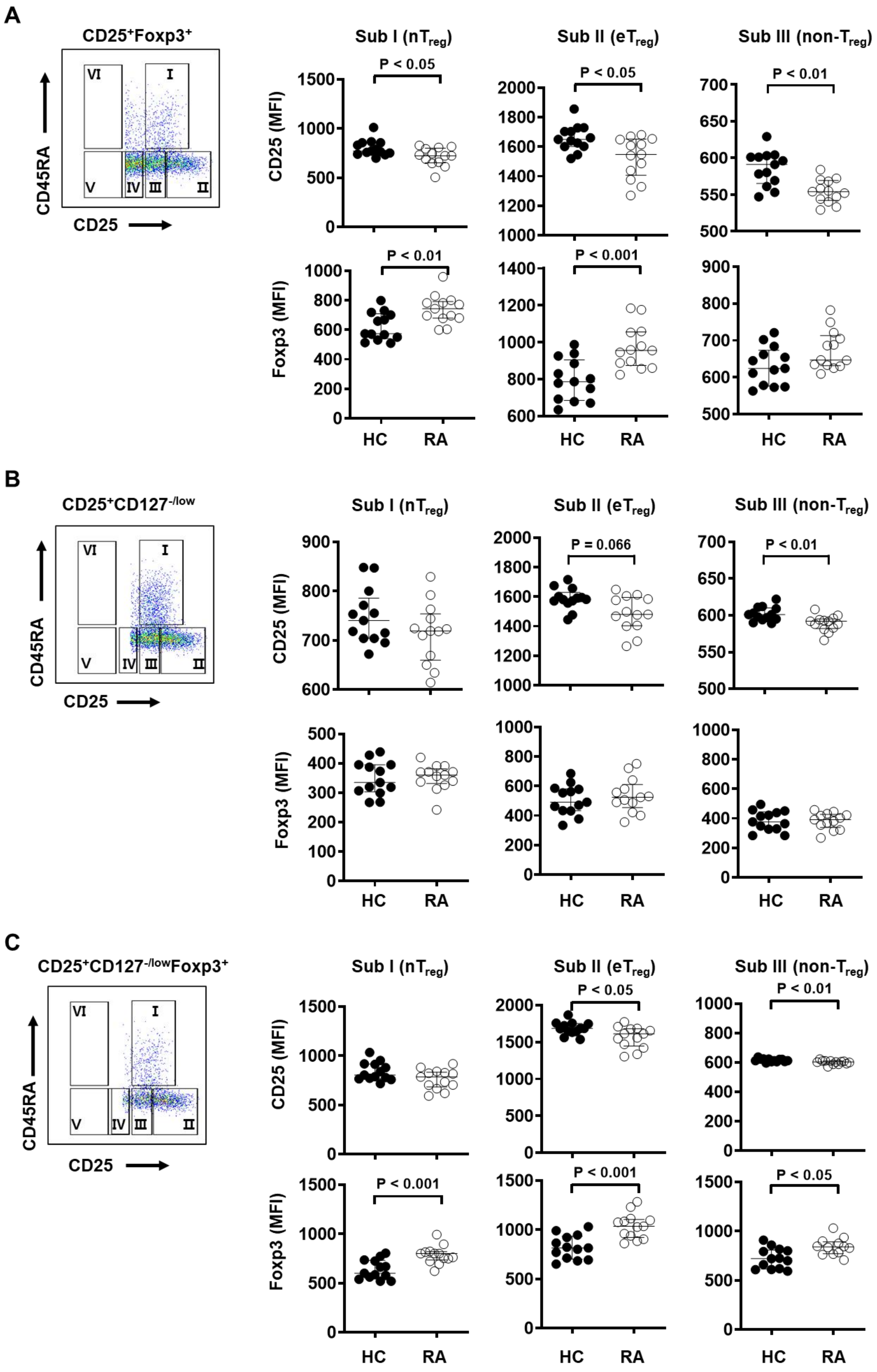


**Figure S3.** Naïve and effector $T_{reg}$ cells from RA patients show decreased CD25 expression. Expression of CD25 and Foxp3 was measured by flow cytometry in $T_{reg}$ cell subgroups I ($CD25^{int}CD45RA^{+}$ cells), II ($CD25^{hi}CD45RA^{-}$ cells), and III ($CD25^{int}CD45RA^{-}$ cells) in PBMCs from healthy controls (HC, n = 13) and RA patients (RA, n = 13). $T_{reg}$ cells were defined as (A) $CD25^{+}Foxp3^{+}$, (B) $CD25^{+}CD127^{-/low}$, or (C) $CD25^{+}CD127^{-/low}Foxp3^{+}$. Statistical differences were calculated by Mann-Whitney test.

**Table S1. Characteristics of Controls and RA subjects**

| Variables | Controls (N = 13) | RA subjects (N = 13) | Unit |
|---|---|---|---|
| Gender | 9 (69.2 %) | 9 (69.2 %) | Female n (%) |
| Age | 43 (26-70) | 52 (22-78) | Median yr (range[1]) |
| Body-mass index[2] | 22.4 (19.4-28.1) | 20.2 (17.7-31.6) | Median (range[1]) |
| Disease activity (DAS28 score) | na[3] | 2.7 (0.5-4.5) | Median (range[1]) |
| Remission (< 2.6) | | 6 (46.2) | N (%) |
| Mild (2.6-3.2) | | 2 (15.4) | N (%) |
| Moderate (3.2-5.1) | | 5 (38.4) | N (%) |
| Severe (>5.1) | | 0 (0) | N (%) |

[1] Minimum to maximum
[2] Body-mass index is the weight in kilograms divided by the square of the height in meters.
[3] not applicable
